# Improving Hip-Worn Accelerometer Estimates of Sitting Using Machine Learning Methods

**DOI:** 10.1249/MSS.0000000000001578

**Published:** 2018-01-26

**Authors:** JACQUELINE KERR, JORDAN CARLSON, SUNEETA GODBOLE, LISA CADMUS-BERTRAM, JOHN BELLETTIERE, SHERI HARTMAN

**Affiliations:** 1 Moores Cancer Center, UCSD, La Jolla, CA;; 2 Department of Family Medicine and Public Health, UCSD, La Jolla, CA;; 3 Children’s Mercy Kansas City, Kansas City, MO; and; 4 Department of Kinesiology, University of Wisconsin–Madison, Madison, WI

**Keywords:** SEDENTARY BEHAVIOR, STANDING, RANDOM FOREST CLASSIFIER, FREE-LIVING

## Abstract

**Purpose:**

This study aimed to improve estimates of sitting time from hip-worn accelerometers used in large cohort studies by using machine learning methods developed on free-living activPAL data.

**Methods:**

Thirty breast cancer survivors concurrently wore a hip-worn accelerometer and a thigh-worn activPAL for 7 d. A random forest classifier, trained on the activPAL data, was used to detect sitting, standing, and sit–stand transitions in 5-s windows in the hip-worn accelerometer. The classifier estimates were compared with the standard accelerometer cut point, and significant differences across different bout lengths were investigated using mixed-effect models.

**Results:**

Overall, the algorithm predicted the postures with moderate accuracy (stepping, 77%; standing, 63%; sitting, 67%; sit-to-stand, 52%; and stand-to-sit, 51%). Daily level analyses indicated that errors in transition estimates were only occurring during sitting bouts of 2 min or less. The standard cut point was significantly different from the activPAL across all bout lengths, overestimating short bouts and underestimating long bouts.

**Conclusions:**

This is among the first algorithms for sitting and standing for hip-worn accelerometer data to be trained from entirely free-living activPAL data. The new algorithm detected prolonged sitting, which has been shown to be the most detrimental to health. Further validation and training in larger cohorts is warranted.

There has been increasing interest in the relationship between sedentary behavior and health (1). Although most studies have shown relationships between *self-reported* measures of sitting time and mortality (2), a few studies have also used sensor-based accelerometer estimates of sedentary behavior and health outcomes such as diabetes and cardiovascular disease (3–5). Most large cohort studies with high-quality prospective follow-up of health outcomes and objective measures have used hip-worn accelerometers because of their ability to accurately measure physical activity (6).

Hip-worn accelerometers have also been used to assess sedentary behavior. A hip-worn accelerometer with an absolute threshold of 100 counts per minute on the vertical axis to assess sitting time is most often used in studies but has also been shown to be problematic (7). Compared with person-worn camera data that can be annotated for different sitting behaviors, the 100-counts per minute cut point has been shown to misclassify standing as sitting and to misclassify sitting while in a vehicle as physical activity (8). Compared with a thigh-worn inclinometer, the hip-worn 100-count threshold has been shown to underestimate standing time and overestimate the number of sit-to-stand transitions (9). Some studies have suggested new cut points for the hip accelerometer, ranging from 25 to 300 counts per minute (10), but such absolute cut points may not always distinguish between sitting still and standing still or moving while sitting and moving while standing (11). Cut points also do not make full use of differences in patterns in the accelerometer signal (beyond counts) across activities, for example, that can be seen in vehicle travel. The presence of errors, however, is cause for concern and could mask or exaggerate relationships with health that have been observed in the large epidemiological studies to date. To improve our understanding and confidence in the findings, better classification techniques for sitting time estimates for the existing hip accelerometer data from the large prospective health studies are needed.

New computation algorithms for hip-worn accelerometers that show promise in improving estimates of physical activity could also perhaps minimize the error seen in the existing cut-point approach to sedentary behavior (12). Many studies developing computational techniques, however, have collected training data for new classifiers in the laboratory setting. Although the laboratory setting allows investigators to observe and control the type of sitting behaviors (e.g., sitting at a desk and sitting in a lounge chair), these behaviors are not performed in free living, and thus, classifiers developed in the laboratory may not perform well in other settings. In particular, laboratory studies have not included sitting in a vehicle, which some populations do for several hours a day. To improve upon such approaches, we have developed behavior classifications based on annotated images from person-worn cameras with up to 12 h of data on multiple days (13). We have shown that such classifiers can detect sitting and standing with greater than 90% accuracy at the minute level. However, when compared with the activPAL, such algorithms only detect 33% of sit–stand transitions, a key sedentary pattern variable, because a transition defines the beginning and end of a sedentary bout.

For specific public health recommendations on sitting, it is important to understand how often we should transition from sitting to standing and what length of sitting bout is related to poor health outcomes. ActivPAL devices, worn on the thigh, detect sit-to-stand transitions by inferring posture from the orientation of the thigh (i.e., thigh is vertical when standing, horizontal when sitting). The accuracy of activPALs has been demonstrated in multiple trials compared with gold standard direct observations with greater than 95% accuracy for sitting (14–17). Although intervention studies have used activPALs to accurately assess changes in sitting time, very few large cohort studies have yet to use this device (6). The infrequent use of activPAL and frequent use of hip-worn accelerometers in large studies warrant the development of new processing methods for hip-worn accelerometers to improve the quality of sedentary behavior and health research. Use of the activPAL as the “ground truth” for algorithm development in detecting postural transitions is beneficial not only because it is valid for this purpose, but also it can be worn for multiple days and hours representing typical free-living behavior and does not depend on a human observer for postural coding. Currently, most algorithms that include sitting classifiers are not trained to specifically detect postural transitions and algorithms or cut points that have been developed from laboratory studies do not include free-living behaviors such as sitting in a vehicle (18,19). Algorithms developed from free-living annotated image data may be limited by the frequency of image capture, which can miss some transitions, and the costs involved in accurate image annotation by human observers may be prohibitive (8). In contrast, the activPAL automatically classifies postures in the standard software package without the need for human observers.

This analysis used a convenience sample of data from 30 breast cancer survivors who concurrently wore a hip-worn accelerometer and a thigh-worn activPAL for 7 d. This study aimed to develop and test a machine learning classifier of posture for the hip-worn accelerometer by using multiple days of the activPAL data as the “ground-truth” training data. We assessed the ability of the classifier to detect sitting, standing, and sit–stand transitions. Because longer bouts of sitting may be worse for health, we also compared estimates of the number of and minutes in sedentary bouts across different sitting bout lengths with the existing 100-counts per minute cut point.

## METHODS

### Participants and Procedures

Breast cancer survivors were enrolled in this cross-sectional pilot study. Eligible participants were women diagnosed with stage I–III breast cancer within the past 5 yr who had completed active treatment (e.g., radiation and chemotherapy) and were fluent in English. Women were excluded if they had a primary or recurrent invasive cancer within the last 10 yr (other than nonmelanomic skin cancer or carcinoma of the cervix *in situ*), were older than 85 yr, recently had bariatric surgery, were taking insulin or corticosteroid medications, or were diabetic. All participants provided written informed consent. Ethical and institutional review board approval for the study was obtained by the University of California, San Diego.

Participants wore the activPAL (PAL Technologies, Glasgow, Scotland), a small and lightweight inclinometer (uniaxial accelerometer) worn on the anterior aspect of the thigh for 24 h for 7 d. Data were processed using activPAL software version 7.2.32. Participants also wore the Actigraph GT3X+ accelerometer for 7 concurrent days but for waking hours only. Raw accelerometer data at 30 Hz were collected on three axes.

We used a postural classification procedure that uses machine learning algorithms to classify the five activPAL postural categories from raw Hertz level triaxial accelerometer data: stepping, standing, sitting, sit-to-stand transition, and stand-to-sit transition. We have developed and tested a similar system to classify activities in three other data sets based on SenseCam images, but the frequency of the image capture may have missed brief sit–stand (13). The classifier is developed using supervised machine learning algorithms: a computational technique that makes use a data set with known labels (i.e., ground truth) to learn associations between features in the data and categories of interest. For this study, we trained the classifier on the current data set of breast cancer survivors, using the matched activPAL postures as the ground truth. We considered the activPAL a suitable ground truth because previous studies have shown the validity of the device and classifier for postural transitions compared with gold standard observations (14–17). Postural categories were assigned every 1 s using the activPAL event file. For the training phase, only 5-s windows with a single postural category were used. For the testing phase, all data were included. Our procedure predicts a posture label (stepping, standing, sitting, sit-to-stand transition, and stand-to-sit transition) for each 5 s of accelerometer data. The posture classification process is composed of three steps: feature extraction, 5-s-level classification, and time filtering. A detailed description of the first two steps can be found in our previous publications (20–22). A short summary is provided here.

#### Feature extraction

The raw (unfiltered) triaxial accelerometer data were split into 5-s windows. For each 5-s window, 41 feature vectors were calculated. For each sample in a data window, the vector magnitude (VM) of the acceleration signal was calculated; that is, *v* = (*x*^2^ + *y*^2^ + *z*^2^)^1/2^. The following basic statistical descriptors of the VM were calculated over the data window: mean; SD (sd); coefficient of variation (coefvariation); minimum (min); maximum (max); and 25th, 50th, and 75th percentiles (25thp, median, 75thp). The 1-s lag autocorrelation (autocorr) of the VM and the correlation between each axis were computed (corrxy, corrxz, corryz). For each sample in the window, the roll, pitch, and yaw angles of the direction of acceleration were computed, as roll = tan^−1^(*y*, *z*), pitch = tan^−1^(*x*, *z*), and yaw = tan^−1^(*y*, *x*). The average (avgroll, avgpitch, avgyaw) and SD (sdroll, sdpitch, sdyaw) of these angles were computed over the window. A low-pass filter with a cutoff frequency of 0.5 Hz (preliminary experiments tested a few cutoff frequencies and found 0.5 Hz to perform best) was applied to the data window to estimate the average direction of gravity, and the roll, pitch, and yaw angles of this direction were computed (rollg, pitchg, yawg) (20). The fast Fourier transform was applied to the VM to decompose the time domain signal to its frequency components. The resulting power spectrum describes the contribution of a given frequency to the measured acceleration signal. The dominant frequency of the signal (fmax), that is, the frequency with the highest power, and corresponding maximal power (pmax) were computed from the power spectrum. A similar calculation was done between the frequency bands of 0.3 and 3 Hz (fmaxband, pmaxband). The entropy of the frequency domain signal was computed. Finally, the power in each frequency band between 1 and 15 Hz (fft1–fft15) was computed.

#### Five-second–level classification

Next, each feature vector was input into a random forest classifier. A random forest classifier is a commonly used machine-learned algorithm made up of an ensemble of randomized decision trees, each of which is learned from a random sample of training data and a random sample of features. The decision tree outputs a probability of each posture label for each feature vector. Chunks of data are classified by averaging the output probabilities from each decision tree in the forest. We used 500 decision trees; each tree is learned from a random sample of 15% of features.

#### Time filtering

After applying the random forest to accelerometer data, a sequence of probabilities of posture labels over time results. Filtering makes use of the probabilities assigned to each activity class by the random forest algorithm. The algorithm first assigns the most probably class to each 5-s window. Then there are two filtering passes. The first pass filters out cases where there are two transitions (sit-to-stand or stand-to-sit) in a row. The 5-s window with the lower probability of transition is reassigned to the next most likely nontransition activity (sit, stand, or step). The second pass filters transitions that do not have the correct activities in the preceding and subsequent 5-s windows (e.g., sit-to-stand preceded by stand and followed by step). These 5-s windows are reassigned to the next most likely nontransition activity (sit, stand, or step). This process removes many false-positive transitions, and we found that it improved overall accuracy. After filtering, a final sequence of postures is obtained by selecting the most likely posture at each point in time.

#### Evaluation

We evaluated the performance of our posture classification algorithms using leave-one-participant-out cross-validation. This means that each participant was used as the test subject in turn, using the remaining participants to train the classification algorithm. Sensitivity, specificity, and balanced accuracy (the mean of sensitivity and specificity) were averaged over each test participant at the 5-s level for each posture (stepping, standing, sitting, sit-to-stand transition, and stand-to-sit transition).

### Statistical Analyses of Bouts

After the machine learning performance testing, outlined previously for the 5-s postural events, additional statistical procedures were used to compare the cut-point and machine-learned methods with the activPAL over different sitting bout durations at the day level. These bout analyses was performed to uncover where the errors in the predictions were occurring and to demonstrate that although total sitting is correlated with existing cut points, there are also errors in bout-level comparison by these methods. To assess the existing cut-point approach, the hip accelerometer data were processed at the minute level in Actilife 6.11, and *a* < 100 counts per minute cut point applied to the vertical axis. Sit–stand transitions were identified as the 1-min epoch at or greater than 100 counts per minute after a sedentary epoch and *vice versa* for stand–sit transitions. The periods between stand–sit and sit–stand transitions were sedentary bouts, and the minutes spent in bouts of various durations were calculated for each day and averaged over all wear days. Wear time was processed using the Choi algorithm in Actilife 6.11, which assesses 90 consecutive minutes of zero *counts* as nonwear and includes a 30-min small window to remove artifactual movement. ActivPAL data for waking hours were matched to the hip accelerometer wear times, with nonwear times from the accelerometer excluded from the activPAL data so that a standard wear time was compared across devices.

Bout durations investigated were <2, 2–5, 5–10, 10–20, 20–30, 30–60, and 60–90 min. Number of daily bouts and total daily minutes accumulated in these bout durations were calculated. Separate generalized estimating equations were used to make comparisons between methods while accounting for clustering within individuals (23). Comparisons were made at the day level. Although these analyses have inherent bias because the machine-learned algorithms were trained on the activPAL data and the cut points were not, the analyses were performed to investigate in which situations (which types of bouts) the algorithms and cut point points performed best at the day level. This provides additional information to the cross-validation at the 5-s level. It was not our aim to develop new cut points from this data set or show that machine learning was a better approach than a trained cut-point approach. We simply wanted to highlight that at the daily bout level there are differences in where misclassification occurs in the standard and new methods.

## RESULTS

Of the 132 women who were contacted about the study, 30 were eligible and completed the clinic visit. The most frequent reason for ineligibility was not being able to commit to study requirements. Participants were a mean (SD) of 62 (8) yr, and 67% of women had been diagnosed with stage 1 breast cancer. As indicated by activPAL ground truth, the average (SD) time spent sitting per day was 499 (83) min, and the average (SD) time spent standing per day was 248 (74) min. There were 51.3 (17.7) sit–stand transitions per day. Average (SD) wear time was 841.4 (54.4) min·d^−1^.

Table 1 shows the performance metrics of the newly developed classifier compared with the activPAL ground truth. The specificity of the postural random forest classifier was higher than its sensitivity.

**TABLE 1:**
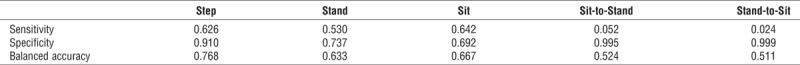
Percent of accurately predicted postures at the 5-s interval from the machine-learned training and testing set using leave-one-out cross-validation.

Table 2 shows the confusion matrix of the classifier for each 5-s epoch, demonstrating that stepping was most often confused with sitting and standing most often confused with sitting. The matrix also shows the small number of transitions that were present in this sample compared with the total number of 5-s epochs spent sitting.

**TABLE 2:**
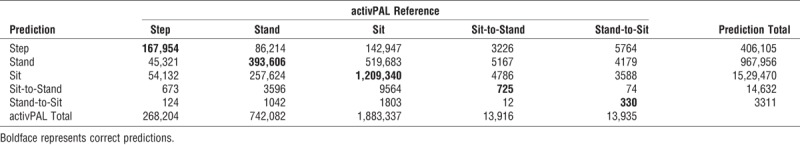
Confusion matrix showing the number of correctly matched 5-s epochs from the activPAL truth and the machine-learned algorithm.

**FIGURE 1:**
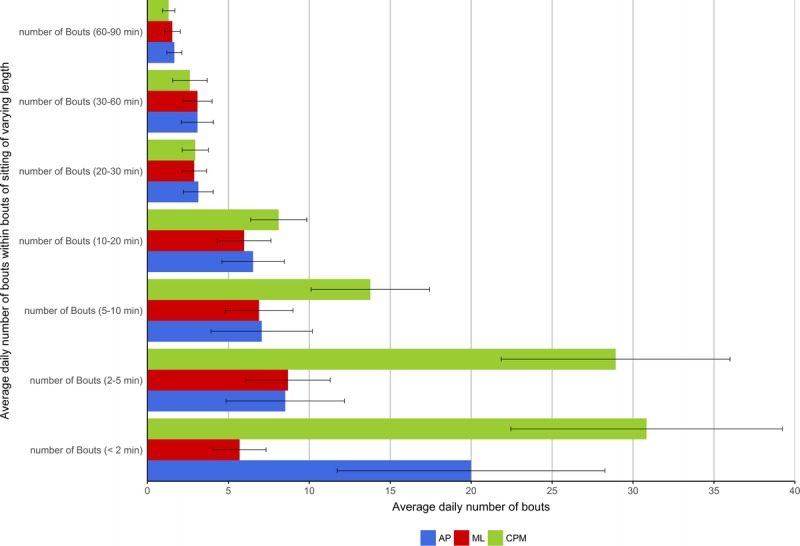
Average number of daily sitting bouts of varying lengths (see Table 3 for significance values). *Error bars represent SD. CPM, counts per minute cut point, ML, machine learning.

**FIGURE 2:**
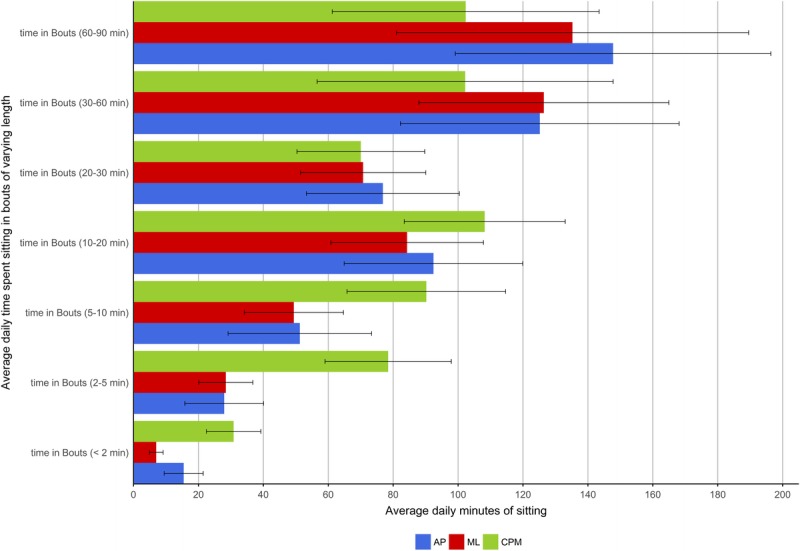
Average total daily time spent in each bout classified as sitting accumulated from bouts of varying lengths (see Table 3 for significance values). *Error bars represent SD. CPM, counts per minute cut point, ML, machine learning.

Because time spent sitting in long or short bouts may be related to health, regardless of total sitting time (24), sitting bout durations were calculated for <2, 2–5, 5–10, 10–20, 20–30, 30–60, and 60–90 min. Number of daily bouts and total average daily minutes accumulated in these bout durations were assessed. Table 3 presents the coefficients and statistical differences between the two approaches and the ground-truth activPAL. The total daily time sitting across devices and methods (using the accelerometer wear time period, in mean (SD)) was as follows: activPAL, 488.00 (126.58); accelerometer machine-learned, 450.00 (139.86), and accelerometer 100-counts per minute cut point, 507.00 (110.13). The average number of bouts in the data set per participant per day identified by the activPAL was 48.6 (17.5). The machine-learned algorithm detected a total of 32.9 (10.4) bouts and the 100-counts per minute cut point detected 87.1 (21.6). When the bouts were classified into bouts lasting <2 min through to bouts lasting 60–90 min, the machine-learned approach only significantly (*P* < 0.001) underestimated the number of bouts lasting <2 min compared with the activPAL from the generalized estimating equation analyses. This indicates where the 5-s errors from the cross validation were most likely to be occurring. The 100-counts per minute approach was significantly (*P* < 0.02–*P* < 0.001) different from the activPAL at all bout durations, but not for total time. Figure 1 illustrates the number of bouts estimated by each approach, and Figure 2 shows the total number of minutes of sitting time averaged across the day from bouts of varying durations. Up to the 10- to 20-min bouts, the 100 counts per minute overestimated the number of bouts and time in bouts; beyond the 20-min bout length, this cut point underestimated bouts and minutes.

**TABLE 3:**
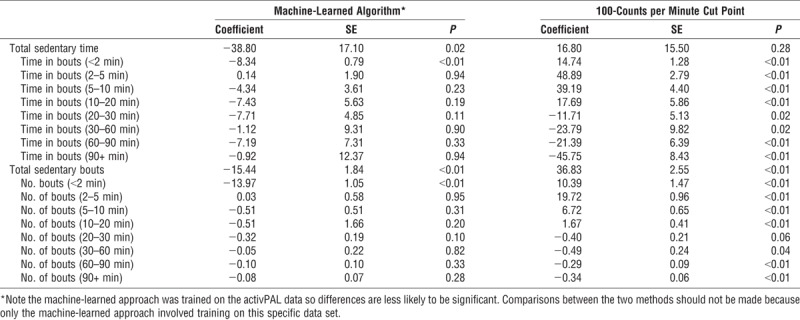
Day-level analyses of number of bouts and minutes in bouts compared by machine learning and standard cut point compared with the activPAL.

## DISCUSSION

This study used the thigh-worn activPAL to provide training data for a machine-learned classifier to detect postural changes from a hip-worn accelerometer. The activPAL has been validated against gold standard observations (14–17) but is not yet commonly deployed in large cohort studies with well-adjudicated health outcomes (6). In these studies, hip-worn accelerometers are most often used, but cut-point approaches mostly used for hip-worn data are problematic for assessing postural transitions (9). Developing a machine-learned classifier from free-living data may improve our estimates of sitting time and transitions for the existing hip-worn accelerometer data in these large cohort studies (25).

The machine learning approach we used was similar to previous studies, but the classifiers’ overall accuracy ranged from only 51% to 77%. This is lower accuracy than achieved in other training data sets (often >80% or 90%) (12,13,26). One reason for this may be that classifier accuracy is related to the number of examples in a training set. In free-living populations, there are few transitions, so the classifier training is limited and the ability to predict accurately is challenged. More balanced data sets can be achieved in the laboratory setting, but then the behaviors do not represent real life. In contrast, laboratory, protocol, and even the free-living image annotation studies only include data when observations are available, which is not always continuous. If observations are not available, the data are excluded. In the activPAL, the postural classification is always available; it does not depend on human observations. When data are excluded in the laboratory or protocols, natural but “messy” transitions are often missed. Such periods are more challenging to predict and may have affected our overall algorithm performance compared with “cleaner” data sets (25).

Despite the overall lower accuracy at the 5-s level, the new machine-learned classifier had very similar estimates to the activPal for number of bouts and number of minutes spent in bouts at the day level. In fact, the only period when the machine-learned classifier performed poorly was in bouts that were less than 2 min long. Further inspection of the data indicated that brief sitting bouts were difficult to detect when they were surrounded by brief bouts of movement, for example, when a participant was frequently getting up and down and moving. Given that the number of transitions was greatest during the period of <2 min (almost 20 from the activPAL; see Fig. 1), this time in particular affected the overall performance of the machine-learned algorithm. It is possible that different features may be able predict this specific behavior, or it may be a limitation of the hip location.

The current analyses show that the cut-point estimates were significantly different from the activPAL, and the direction of the relationship changed depending on the time in bouts. It overestimated number and time in bouts less than 20 min and underestimated the time and number of prolonged bouts greater than 20 min. This is important because many studies use the cut point to determine total sitting time or breaks from sitting time (3,4). The overall number of sit–stand transitions was also significantly greater, almost double the activPAL estimates. In contrast, the total minutes of sitting time was not significantly different. The differences in total and bout-related estimates indicate that comparisons of total sitting may be hiding important underlying differences.

This study was limited by a small sample of breast cancer survivors, and results may not be generalizable. We caution researchers applying algorithms from laboratory studies or a specific population to more general population studies of free-living individuals (22). Replication of the performance testing in larger independent cohorts is needed (27). Given that the public health evidence points toward the importance of prolonged bouts (30+ min) (3,4), where this classifier performed best, we believe that this type of classifier may provide accurate measurement of sitting in the large cohort studies of hip accelerometer data with health outcomes. Further training in larger populations with more examples of transitions or combining free-living and laboratory training data may improve the algorithm. Future studies could include estimates of vehicle time and explore intensity of the movement to inform the algorithm development. New features to better capture brief transitions and machine learning approaches such as recurrent neural networks (which are not tied to a specific time window) may improve algorithm performance. We believe that an algorithm can be sufficiently developed to address previous cut-point limitations and allow sitting time to be estimated in the hip location with sufficient accuracy for epidemiological association studies. This study provides a proof of concept that an algorithm can be developed from an activPAL to detect sitting time on a hip-worn accelerometer. Further training in larger samples, validation in independent samples, and applications with health outcomes will progress this field.
